# Leptospirosis with acute renal failure and paraparesis

**DOI:** 10.4103/0971-4065.43695

**Published:** 2008-07

**Authors:** P. Ramakrishna, V. V. Sai Naresh, B. Chakrapani, B. Vengamma, V. Siva Kumar

**Affiliations:** Department of Nephrology, Sri Venkateswara Institute of Medical Sciences, (SVIMS) Tirupati, India; 1 Department of Neurology, Sri Venkateswara Institute of Medical Sciences, (SVIMS) Tirupati, India

**Keywords:** Leptospirosis, acute renal failure, paraparesis

## Abstract

Leptospirosis is an important zoonosis with a worldwide distribution that is characterized by a broad spectrum of clinical manifestations ranging from inapparent infection to fulminant disease. The presentation of paraparesis in combination with acute renal failure is rare.

## Introduction

Leptospirosis is a zoonosis caused by pathogenetic leptosires. It presents with a wide spectrum of clinical manifestations ranging from inapparent infection to fulminant and fatal disease. The spirochetes are transmitted from direct contact with the urine, blood, or tissue of infected rodents. After an incubation period of 1–2 weeks, leptospirosis manifests as a biphasic illness with the leptospiremic phase followed by an immune phase. The clinical spectrum of the disease may be an influenza-like fever to a serious presentation such as Weil's syndrome characterized by hepatic, renal, neurological, and hematological abnormalities. Leptospirosis is a worldwide disease with a predominance in tropical, rural areas and its presentation in combination with paraparesis and acute renal failure is uncommon.^1^,^2^

## Case Report

A 26 year-old female patient presented a week postpartum with a history of fever, diarrhea of three days' duration, oliguria, edema of the legs, and bilateral lower limb motor weakness of one day's duration with no sensory or bladder involvement. There was no past history of diabetes, hypertension, or any similar illness. Clinical evaluation revealed pitting edema legs, normotension (blood pressure 130/80 mm Hg), paraparesis (grade 2/5 motor power), and no sensory or bladder abnormalities. Examination of the higher functions, cranial nerves, and cerebellar systems did not reveal any remarkable findings; there were no meningeal signs. Urine analysis showed 2+ proteinuria, a few RBCs, and occasional pus cells. The hemoglobin level was 12.5 g/dL, total leukocyte count was 6800 cells/cubic mm, and the platelet count was 75000/cubic mm. Renal function tests revealed severe renal failure (blood urea: 113 mg/dL, serum creatinine: 4 mg/dL, sodium: 159 meq/L, potassium: 6.9 meq/L). Liver function tests showed a total bilirubin: 0.5 mg/dL; direct bilirubin: 0.1 mg/dL, aspartate aminotransferase: 201 U/L, alanine aminotransferase: 158 U/L, alkaline phosphatase: 53 U/L, serum total protein: 5.2 g/dL, and albumin: 2.7 g/dL. Also observed were corrected serum calcium: 8.8 mg/dL, phosphorus: 4.5 mg/dL, serum CPK: 570 U/L, and serum lactate dehydrogenase: 112 U/L. Urine and blood cultures were sterile. Serum IgM antibodies for leptospira could be detected by enzyme linked immunoassay and the microagglutination test (MAT test) revealed significant antibody titers for *Leptospira australis* (1 in 100 titre). The kidneys were echogenic and bulky on ultrasonography while computed tomography of the brain showed an old infarct in the right frontoparietal area. Magnetic resonance imaging of the spinal cord was normal; nerve conduction studies were suggestive of bilateral crural radiculopathy.

Based on the above findings, the possibility was considered of leptospirosis manifesting with acute renal failure and paraparesis. The patient improved with antibiotics, hemodialysis, and physiotherapy support. She was discharged in an ambulatory state with improved renal function (serum creatinine: 1.2 mg/dL).

## Discussion

Leptospirosis is a worldwide zoonosis of great public health importance in the tropics. Infection may be asymptomatic but can be fatal in 5–15% of all cases being associated with hepatic, renal, neurological, and hematological abnormalities. Common presentations in neuroleptospirosis are asymptomatic meningitis and encephalitis; paraparesis due to myelitis or radiculopathy is very rare.^2^,^3^

This patient presented with sudden onset paraparesis and acute renal failure with hyperkalemia preceded by a febrile illness with diarrhea. Serological investigations revealed the presence of IgM antibodies against leptospira and significant antibody titers (1 in 100) against *Leptospira australis* in the MAT test. The patient improved with antibiotics and dialysis support, and was discharged in an ambulatory state with improved renal function. In reviewing the cause of the paraparesis (leptospirosis *vs* hyperkalemia), it may be logical to conclude that the paraparesis was secondary to the leptospirosis because of the slow improvement in motor function of both lower limbs despite the early correction of hyperkalemia by hemodialysis.
